# Rare Vascular Sarcoma Treated With Ex Vivo Liver Surgery: A Case of IVC Leiomyosarcoma

**DOI:** 10.1155/cris/6854083

**Published:** 2026-06-16

**Authors:** Amirhassan Rabbani, Hesameddin Eghlimi, Mina Khoshkbar Foroushan, Mahmoud Amiri

**Affiliations:** ^1^ Department of General Surgery, School of Medicine, Ayatollah Taleghani Hospital, Shahid Beheshti University of Medical Sciences, Tehran, Iran, sbmu.ac.ir; ^2^ Department of Medical-Surgical Nursing, School of Nursing and Midwifery, University of Medical Sciences, Tehran, Iran, tums.ac.ir

**Keywords:** inferior vena cava reconstruction, leiomyosarcoma of the inferior vena cava, liver autotransplantation

## Abstract

We report a case of leiomyosarcoma of the inferior vena cava (IVC) treated with liver resection, autotransplantation, and IVC reconstruction using a Dacron graft. A 47‐year‐old female presented with mild to moderate lower extremity edema and weakness. Physical examination revealed no abdominal or pelvic abnormalities. However, further evaluation confirmed a tumor in the suprarenal IVC, extending to the hepatic veins, with thrombus formation in the suprahepatic IVC. During surgery, the tumor was confirmed to originate from the IVC. Given its proximity to the hepatic veins, an ex vivo liver resection and autotransplantation (ELRA) were performed, followed by IVC reconstruction using a Dacron graft via the piggyback technique. The patient experienced no postoperative complications. Although liver resection and autotransplantation are considered extremely aggressive procedures, they can be viable options in cases where complete surgical resection is not feasible using standard techniques.

## 1. Introduction

Tumors of the inferior vena cava (IVC) are rare and most commonly malignant, arising either as primary vascular tumors or from secondary invasion. The most frequent primary IVC tumor is leiomyosarcoma, originating from the smooth muscle layer of the vessel wall [1]. Due to its insidious growth and nonspecific clinical presentation, diagnosis is often delayed until an advanced stage [2].

The mainstay of treatment is complete surgical resection with negative margins, which remains the only potentially curative option. However, optimal surgical strategies and the role of adjuvant therapy remain controversial due to the rarity of this disease [3].

In selected cases of retrohepatic IVC tumors with extensive cranial extension approaching the hepatic veins, standard in situ resection may not be feasible, and advanced surgical strategies such as liver resection with ex vivo repair and autotransplantation may be required as a salvage approach [4].

## 2. Case History

A 47‐year‐old woman from Iran presented with a 3‐month history of progressive weakness and bilateral lower extremity swelling. She had no past medical history, comorbidities, or family history of malignancy. Physical examination revealed mild to moderate bilateral pitting edema of the lower extremities.

Laboratory investigations demonstrated anemia with a hemoglobin level of 8.9 g/dL and elevated liver transaminases (AST: 436 U/L, ALT: 319 U/L).

**Figure 1:**
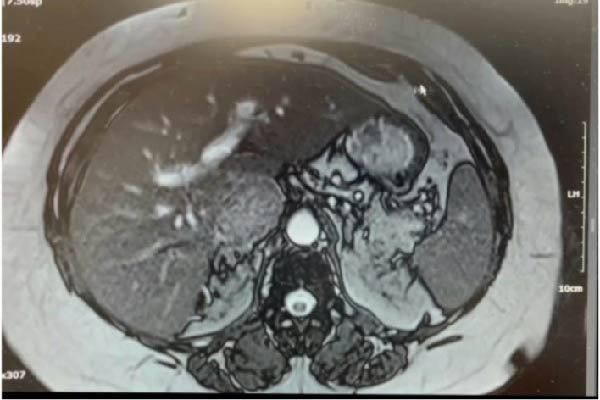
Cross‐section MRI showed lesion in IVC.

Abdominal ultrasonography revealed a hypoechoic retroperitoneal mass arising from the infrahepatic IVC. Subsequent contrast‐enhanced magnetic resonance imaging (MRI) demonstrated a 10 × 5 cm heterogeneous mass involving the infrahepatic IVC with complete luminal obstruction (Figure 1). In addition, an 8.9 × 4 cm intraluminal tumoral extension was identified, extending cranially toward the intrahepatic IVC.

Based on imaging characteristics and clinical correlation, a primary IVC tumor was strongly suspected, with leiomyosarcoma considered the most likely diagnosis.

## 3. Treatment and Outcome

Due to the cranial extension of the tumor up to the level of the hepatic veins, tumor resection and IVC reconstruction were technically challenging. Therefore, an ex vivo liver resection and autotransplantation (ELRA) approach was selected to facilitate safe tumor removal and to avoid prolonged hepatic ischemia that could result from extended clamping of the hepatic veins and suprahepatic IVC.

**Figure 2:**
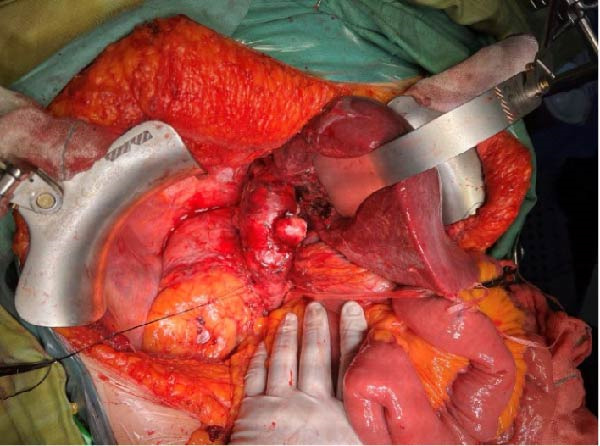
Tumoral lesion of infrahepatic IVC.

A laparotomy was performed through a Mercedes incision. Intraoperative exploration revealed a large mass arising from the IVC, extending from the suprarenal segment up to the confluence of the hepatic veins (Figure 2). There was no evidence of invasion into adjacent structures, including the aorta, kidneys, or duodenum. No suspicious lymphadenopathy, liver lesions, peritoneal seeding, or ascites were identified.

Following dissection of the hepatic hilum, vascular inflow and outflow structures were isolated. The liver was then explanted to allow ex vivo tumor resection. Control of the IVC was achieved proximally near the right atrium and distally above the renal veins. The diaphragm was partially dissected to facilitate adequate exposure and vascular control.

**Figure 3:**
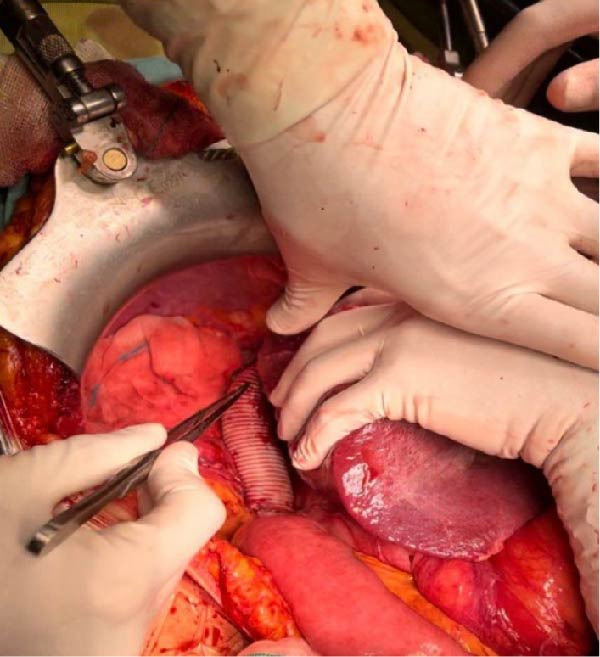
IVC reconstruction with prosthetic graft.

During the bench procedure, the explanted liver was flushed with University of Wisconsin (UW) preservation solution to minimize ischemic injury. The tumor‐bearing segment of the IVC was completely resected. Reconstruction of the IVC was performed using a 150‐mm Dacron graft, restoring caval continuity (Figure 3).

Subsequently, liver autotransplantation was carried out using the piggyback technique. Hepatic venous outflow was reconstructed by anastomosing the preserved hepatic veins to the reconstructed IVC. Portal vein and hepatic artery continuity were restored via end‐to‐end anastomoses. Biliary reconstruction was performed using a duct‐to‐duct anastomosis.

After completion of vascular and biliary reconstruction, adequate graft perfusion was confirmed, and meticulous hemostasis was achieved.

**Figure 4:**
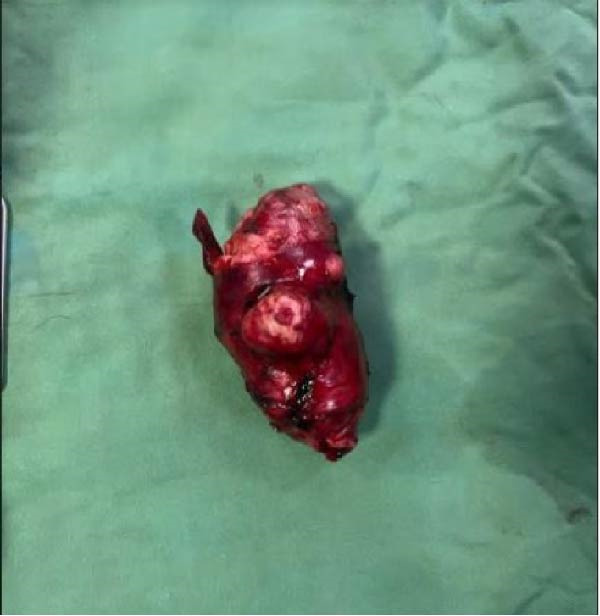
En‐bloc resection of tumorous IVC.

**Figure 5:**
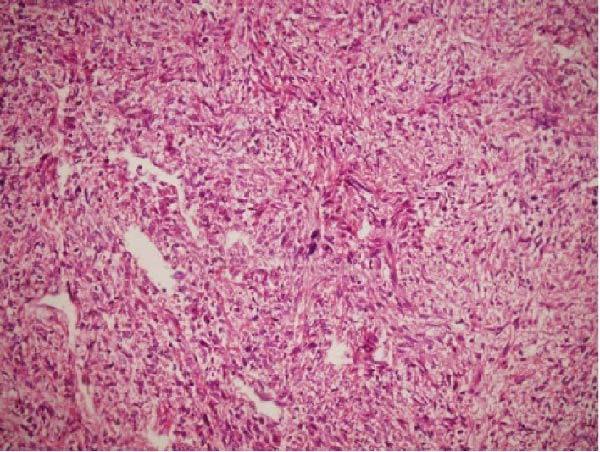
H&E stained.

**Figure 6:**
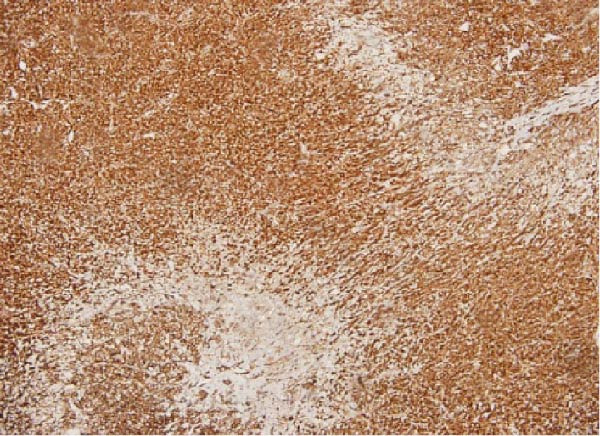
IHC pathological tissue section.

Histopathological examination of the resected specimen revealed a partially encapsulated, creamy‐brown nodular mass measuring 10 × 6 × 4 cm with firm consistency on gross evaluation (Figure 4). Microscopically, the tumor was composed of intersecting fascicles of spindle‐shaped cells with elongated, blunt‐ended (“cigar‐shaped”) nuclei and eosinophilic cytoplasm, consistent with smooth muscle differentiation (Figure 5). Moderate nuclear atypia was observed. Mitotic activity was increased, with a rate of 10–19 mitoses per 10 high‐power fields (HPF) (score 2). Tumor necrosis was present in less than 50% of the tumor area (score 1). Immunohistochemical (IHC) analysis demonstrated diffuse positivity for H‐caldesmon and smooth muscle actin (SMA), supporting smooth muscle origin. The tumor cells were negative for desmin, S100, CD31, and CD34. The Ki‐67 proliferation index was greater than 10% of tumor cells, indicating moderate proliferative activity (Figure 6). Based on the histomorphological and IHC findings, the tumor was diagnosed as conventional leiomyosarcoma arising from the IVC. According to the FNCLCC grading system, the tumor was classified as Grade 2, with a differentiation score of 2, and surgical margins were free of tumor (R0 resection). The patient’s postoperative course was uneventful, and she subsequently received adjuvant chemoradiotherapy following multidisciplinary evaluation. At 1‐year follow‐up, she remains clinically well with no evidence of recurrence.

## 4. Discussion

Primary leiomyosarcoma of the IVC is a rare vascular malignancy arising from the smooth muscle layer of the vessel wall. Due to its slow growth and nonspecific clinical presentation, it is often diagnosed at an advanced stage. In some cases, tumor extension into adjacent vascular structures, including the renal veins, hepatic veins, or even the right atrium, may occur, leading to impaired hepatic or renal function and further complicating management [5, 6]. Despite these challenges, complete surgical resection with negative margins remains the only potentially curative treatment, as these tumors demonstrate limited responsiveness to chemotherapy and radiotherapy [2, 7].

IVC leiomyosarcomas are commonly classified based on anatomical location into Level 1 (infrarenal), Level 2 (pararenal, suprarenal, and infrahepatic), and Level 3 (suprahepatic), with Level 2 being the most frequently encountered [1, 8–10]. Given the complexity and variability in tumor location, a multidisciplinary approach is essential for optimal surgical planning and outcomes [11]. Tumors involving the retrohepatic IVC, particularly those extending toward the hepatic vein confluence, present significant technical challenges. In such cases, conventional in situ resection may be limited by inadequate exposure and the need for prolonged clamping of the suprahepatic IVC and hepatic veins. This can result in hepatic congestion, impaired venous outflow, and warm ischemic injury, thereby increasing the risk of postoperative liver dysfunction.

In the present case, the tumor originated from the suprarenal and infrahepatic segments of the IVC (Level 2) and extended cranially toward the hepatic vein confluence. This anatomical proximity made safe in situ resection technically unfeasible, as adequate vascular control would have required prolonged suprahepatic IVC and hepatic vein clamping, posing a significant risk of hepatic injury. Therefore, an ELRA approach was selected. This strategy allowed complete explantation of the liver, providing optimal exposure for precise tumor resection and facilitating controlled vascular reconstruction. In addition, cold perfusion of the liver minimized ischemic injury and enabled safe reconstruction of the IVC using a prosthetic graft, which is a well‐established technique in cases requiring extensive venous resection [1, 12].

ELRA offers several advantages over conventional approaches, including improved visualization, enhanced vascular control, and the ability to perform meticulous tumor resection and reconstruction under controlled conditions [13, 14]. Although initially developed for complex benign conditions such as end‐stage hepatic alveolar echinococcosis [15–17], this technique has increasingly been applied in selected malignant cases, particularly in tumors deemed unresectable due to proximity to critical vascular structures [18]. Furthermore, its application has been reported in a limited number of cases involving IVC leiomyosarcomas with intrahepatic or retrohepatic extension [2, 19]. Despite its advantages, ELRA remains a highly complex and technically demanding procedure that should be reserved for carefully selected patients and performed in specialized centers with appropriate expertise.

This case highlights the importance of individualized surgical planning in patients with IVC leiomyosarcoma. In selected cases with tumor extension toward the hepatic venous outflow, ELRA may represent a feasible and effective strategy to achieve complete resection while minimizing hepatic injury.

Given the tumor’s large size, intermediate histologic grade, and anatomically complex location within the IVC, the risk of recurrence was considered significant despite complete (R0) resection. Following multidisciplinary tumor board discussion, an individualized decision was made to administer adjuvant chemoradiotherapy. At 1‐year follow‐up, the patient remains clinically well, with no evidence of local recurrence or distant metastasis.

## 5. Conclusion

Leiomyosarcoma of the IVC is a rare tumor that poses significant challenges due to its location, making surgical resection complex. However, radical surgical resection remains a potential curative treatment option. Ex vivo liver resection combined with autotransplantation offers a viable approach for achieving complete resection with negative margins, providing hope for improved outcomes in such difficult cases.

## Funding

No funding was received for this study.

## Conflicts of Interest

The authors declare no conflicts of interest.

## Data Availability

The data that support the findings of this study are available upon request from the corresponding author. The data are not publicly available due to privacy or ethical restrictions.

## References

[1] Maniso F. H. , Woldegeorgis M. A. , and Bedada H. F. , Surgical and Oncologic Approach to Leiomyosarcoma of the Inferior Vena Cava: A Case Report, Clinical Case Reports. (2024) 12, no. 8. 10.1002/ccr3.9336 PMC11335577 39171334

[2] Tuxun T. , Li T. , and Apaer S. , et al.Ex Vivo Liver Resection and Autotransplantation as a Surgical Option for Zone II–III Leiomyosarcoma of the Inferior Vena Cava: A Case Report and Literature Review, Frontiers in Oncology. (2021) 11. 10.3389/fonc.2021.690617 PMC8226245 34178689

[3] Davidovic L. , Ducic S. , and Dragas M. , et al.Case Series of Inferior Vena Cava Primary Leiomyosarcoma Treatment, Journal of Surgical Case Reports. (2024) 2024, no. 6, 10.1093/jscr/rjad546, rjad546. 38840898 PMC11151786

[4] Rahnemai-Azar A. A. , Griesemer A. D. , Velasco M. L. , and Kato T. , Ex Vivo Excision of Retroperitoneal Soft Tissue Tumors: A Case Report, Oncology Letters. (2017) 14, no. 4, 4863–4865, 10.3892/ol.2017.6797. 29085493 PMC5649545

[5] Mann G. N. , Mann L. V. , Levine E. A. , and Shen P. , Primary Leiomyosarcoma of the Inferior Vena Cava: A 2-Institution Analysis of Outcomes, Surgery. (2012) 151, no. 2, 261–267, 10.1016/j.surg.2010.10.011. 21176932

[6] Cananzi F. C. , Mussi C. , and Bordoni M. G. , et al.Role of Surgery in the Multimodal Treatment of Primary and Recurrent Leiomyosarcoma of the Inferior Vena Cava, Journal of Surgical Oncology. (2016) 114, no. 1, 44–49, 10.1002/jso.24244. 27062161

[7] Dull B. Z. , Smith B. , Tefera G. , and Weber S. , Surgical Management of Retroperitoneal Leiomyosarcoma Arising From the Inferior Vena Cava, Journal of Gastrointestinal Surgery. (2013) 17, no. 12, 2166–2171, 10.1007/s11605-013-2385-0. 24146340 PMC3838601

[8] Ameeri S. , Butany J. , and Collins M. J. , et al.Leiomyosarcoma of the Inferior Vena Cava, Cardiovascular Pathology. (2006) 15, no. 3, 171–173, 10.1016/j.carpath.2005.08.011. 16697934

[9] Kulaylat M. N. , Karakousis C. P. , Doerr R. J. , Karamanoukian H. L. , O’Brien J. , and Peer R. , Leiomyosarcoma of the Inferior Vena Cava: A Clinicopathologic Review and Report of Three Cases, Journal of Surgical Oncology. (1997) 65, no. 3, 205–217. 9236931 10.1002/(sici)1096-9098(199707)65:3<205::aid-jso11>3.0.co;2-2

[10] Kieffer E. , Alaoui M. , Piette J.-C. , Cacoub P. , and Chiche L. , Leiomyosarcoma of the Inferior Vena Cava: Experience in 22 Cases, Annals of Surgery. (2006) 244, no. 2, 289–295, 10.1097/01.sla.0000229964.71743.db. 16858193 PMC1602179

[11] Chellasamy R. T. , Sivanesan A. , Kalyanasundaram A. , Munusamy H. , and Rajarajan N. , Approach to Different Types of Inferior Vena Cava Leiomyosarcomas: A Vascular Surgeon’s Perspective, Cureus. (2023) 15, no. 6, 10.7759/cureus.40694. PMC10358784 37485149

[12] Kaneko J. , Hayashi Y. , and Kazami Y. , et al.Resection and Reconstruction of the Inferior Vena Cava, Hepatic, and Portal Veins: A Narrative Review, Translational Gastroenterology and Hepatology. (2024) 9, 10.21037/tgh-23-90, 23. 38716218 PMC11074493

[13] Li W. , Zhang X. , Zhang Y. , and Wang R. , Management of Primary Inferior Vena Cava Leiomyosarcoma: A Comprehensive Case Study, Frontiers in Oncology. (2023) 13, 10.3389/fonc.2023.1190276, 1190276. 38023228 PMC10680028

[14] Zhou X. , Wang M. , and Li S. , et al.A Case of a Huge Inferior Vena Cava Leiomyosarcoma: Precise Preoperative Evaluation With Gadobutrol-Enhanced MRI, Cancer Management and Research. (2020) 12, 7929–7939, 10.2147/CMAR.S258990. 32943927 PMC7473983

[15] Humaerhan J. , Jiang T. M. , Aji T. , Shao Y. M. , and Wen H. , Complex Inferior Vena Cava Reconstruction During Ex Vivo Liver Resection and Autotransplantation: A Case Report, World Journal of Clinical Cases. (2023) 11, no. 23, 5602–5609, 10.12998/wjcc.v11.i23.5602. 37637699 PMC10450365

[16] Ruze R. , Jiang T. M. , and Zhang W. , et al.Liver Autotransplantation and Atrial Reconstruction in Multiorgan Alveolar Echinococcosis: A Case Report, BMC Infectious Diseases. (2024) 24, no. 1, 10.1186/s12879-024-09545-0, 659. 38956482 PMC11218102

[17] Lin X. , Shao Y. M. , Zhang R. Q. , and Aji T. , Prediction of Biliary Complications After Ex Vivo Liver Resection and Autotransplantation, European Journal of Medical Research. (2024) 29, no. 1, 10.1186/s40001-024-01898-1, 301. 38812045 PMC11134669

[18] Yang X. , Lu L. , and Zhu W. W. , et al.Ex Vivo Liver Resection and Autotransplantation for Liver Malignancies: Progress and Challenges, Hepatobiliary & Pancreatic Diseases International. (2024) 23, no. 2, 117–122, 10.1016/j.hbpd.2023.10.007. 38619051

[19] Foguenne M. , Marique L. , and Coubeau L. , Ex Situ Liver Resection and Autotransplantation With Retrohepatic Inferior Vena Cava Reconstruction and Atrial Thrombectomy Under Extracorporeal Circulation for Inferior Vena Cava Leiomyosarcoma, Annals of Surgical Oncology. (2024) 31, no. 10, 7206–7207, 10.1245/s10434-024-15622-0. 38926212

